# The impact of the Covid-19 pandemic on maternal delivery experiences and breastfeeding practices in China: data from a cross-sectional study

**DOI:** 10.1186/s12887-022-03155-y

**Published:** 2022-02-24

**Authors:** Jinyue Yu, Mingyue Gao, Zhuang Wei, Jonathan C. K. Wells, Mary Fewtrell

**Affiliations:** 1 Population, Policy & Practice Research & Teaching Department, UCL Institute of Child Health, University College London, London, UK; 2 Department of Child Health, Capital Medical University Beijing Children’s Hospital, Beijing, China

**Keywords:** COVID-19, Birth, Infant feeding, Postpartum, Breastfeeding

## Abstract

**Background:**

The COVID-2019 pandemic has placed extensive pressure on health systems and posed a severe public health challenge worldwide. Lockdown measures implemented in many countries have delayed virus spread. However, a considerable number of people have faced unprecedented pressure, especially pregnant and breast-feeding women, because face-to-face professional support has been reduced during the lockdown in many countries.

**Objectives:**

To compare the delivery and infant feeding experiences of women who delivered before (BL) versus during (DL) the Covid-19 pandemic in Beijing, China and to investigate predictors of breastfeeding at 6-months.

**Methods:**

Women aged ≥18 years with an infant ≤18 months of age completed an anonymous survey. Information/links were shared online and via local clinics in Beijing. Logistic regression was performed to assess predictors of breastfeeding during the first 6-months.

**Results:**

One thousand eight hundred seven women provided data; BL 1231 (68.1%), DL 576 (31.9%). Significantly more mothers in DL group reported the lockdown had moderate to high impact to their household income (*p* = 0.013) and the convenience of purchasing daily necessities(*p* = 0.014). Compared to BL mothers, significantly more mothers in the DL groups thought their birth location and breastfeeding intention had been effected by the COVID-19 (*p* < 0.001, *p* = 0.036 respectively). Mostly breastfeeding (MBF, mainly breastfeeding with few non-formula fluids added) at 6 months was predicted by delivery during the lockdown period (OR1.43, 95% confidence interval (CI) 1.08, 1.90), younger maternal age (OR 0.96, 95%CI 0.93, 0.99), getting support from friends or relatives (OR 1.95, 95%CI 1.06, 3.59), and discussing health issues in online groups > four times a week (OR 1.66, 95%CI 1.09, 2.53).

**Conclusion:**

The COVID-19 pandemic and lockdown measures influenced mothers’ planned birth location and breastfeeding intention. However, breastfeeding practice was maintained during the pandemic. Our results highlight the importance of feeding support as well as potential beneficial effects of increased mother-infant contact during the lockdown period which is relevant even under normal circumstances.

**Supplementary Information:**

The online version contains supplementary material available at 10.1186/s12887-022-03155-y.

## Synopsis

**Study question:** What impact did the pandemic have on delivery experience and infant feeding and are there practical measures that can be identified to improve maternal experiences and infant feeding?

**What’s already known**: Previous studies showed breastfeeding can be affected by changes of maternal social-economic status, supports, and delivery experiences that influenced by the COVID-19 pandemic, and the impact may vary among countries.

**What this study adds:** The study assessed the relationship between lockdown measures during the COVID-19 pandemic and their impacts on maternal delivery and feeding practice in Beijing, China. Results suggested that the CPVID-19 pandemic had affected mothers’ planned birth location and breastfeeding for the first 6 months of birth. Notably, we found mothers with younger age, getting support from friends or relatives compared to no support, and chatting in online maternal supporting group more than 4 times a week were more likely to breastfeeding their infants.

## Introduction

The coronavirus disease 2019 (COVID-2019) pandemic, caused by severe acute respiratory syndrome coronavirus 2 (SARS-CoV-2), began in December 2019 and has placed extensive pressure on health systems and posed a severe public health challenge worldwide [1, 2]. COVID-19 affects people of all ages, with no significant gender difference [3–5]. Lockdown measures, including closure of schools, workplaces and other public places, were implemented in many countries and have delayed virus spread [6]. Nevertheless, confronted with the COVID-19 pandemic and pervasive disruption to daily life,many people have faced unprecedented pressure. This is particularly the case for pregnant and breast-feeding women, because face-to-face professional support has been reduced during the lockdown in many countries.

Breast milk is the optimum food for infants, and provides the energy and essential nutrients required during the first few months of life as well as non-nutritive bioactive components, many of which provide protection against infection [2]. A systematic review including 49 studies found no association between infection of COVID-19 and feeding method or maternal proximity [7]. The World Health Organization (WHO) also recommended that mothers with COVID-19 (or suspected COVID-19) should breastfeed as long as they take appropriate precautions [4, 8]. However, the lockdown measures undertaken in several countries to prevent the spread of virus impacted maternal postpartum experiences, leading to distress for a large number of women and affecting their breastfeeding practice [9–11].

In Beijing, China, general lockdown measures were in place between from 26th January to 31st July 2020 [12]; most public places apart from essential stores were closed. Travel restrictions were imposed and meeting with those from other households limited, other than for caring or work purposes. Additionally, as a means of ensuring the safety of mothers and babies, routine postpartum home visits were replaced with telephone consultations, although mothers were able to request face-to-face appointments if required, following a triage process to identify suspected cases of COVID-19. Although these strategies effectively protected postpartum women from infection, the impact of such strict policies on women’s wellbeing and breastfeeding practice remains unknown. Studies show that postnatal experiences and breastfeeding support are strongly associated with breastfeeding initiation and duration [13–15]. Given that breastfeeding is optimally supported through high-quality professional and peer-to-peer support and positive maternal wellbeing [16], and the COVID-19 pandemic is ongoing in a large majority of countries, it is important to understand the impact of the pandemic and lockdown measures on women’s ability to breastfeed.

This study therefore aimed to investigate: 1) The delivery experience, feeding intentions and actual feeding methods of mothers who delivered before or during the lockdown; 2) The impact of lockdown on breastfeeding practice and support; 3) Factors that predict infant feeding methods in the first 6 months. The research is important to identify problems experienced by this vulnerable group, and will also contribute to the formulation of public health policies as the current COVID-19 pandemic continue, as well as in future public health emergencies.

## Methods

### Recruitment

The research was undertaken between 1st August 2020 and 31st October 2020, at a number of local clinics attached to Beijing Children’s Hospital (BCH) and located in different districts in Beijing, China. Advertisements, including a brief introduction to the research and the inclusion criteria, were posted on the bulletin board in each local clinic’s reception area. The inclusion criteria were: 1) mother ≥18 years; 2) infant aged between 6 months and 18 months at the time of survey completion; 3) living in Beijing and breastfeeding their infant (exclusively or partially) for some or all of the lockdown period (during 26th January to 31st July 2020); 4) mother had no complications or other chronic disease during the pregnancy or postnatal period. Eligible mothers were invited to complete a one-time, anonymous questionnaire, which consisted of 48 questions and took approximately 15–20 min to complete. The questionnaire was provided both online and in a paper version and mothers could choose which one they wanted to complete. The online version was supported by the WenjuanNet (https://wenjuan.net/s/NVveyew/), which can create online questionnaire with a large number of templates provided. A QR code was generated when the online survey started. Eligible mothers who were interested in participation could begin the survey on their smart phone by scanning the QR code, or by clicking the invitation link.

Respondents were informed that their participation was voluntary and their consent to this study was implied by completion of the questionnaire. The first page of the survey provided information about the study. Mothers could complete the questionnaire during their waiting time in the clinic, and a trained nurse was available to assist the mothers in completing the questionnaire. We finally retrieved 1807 eligible mother’s information. The response rate for each surveyed question ranged 90–100% (as shown in Tables 1, 2 and 3). Two investigators imported the raw data into SPSS separately. If there were discrepancies, they both would double-check the original questionnaire and correct the potential errors. Data from the online questionnaire were automatically exported to the SPSS.

**Table 1:** Background characteristics of mothers who completed the survey (mean ± SD / n(%))

Mean ± SD / N(%)	N (%)	Before	During	Total	***P*** value
		(***N*** = 1231)	(***N*** = 576)	(***N*** = 1807)	
Maternal age (years)	1771 (98)	31.6 ± 4.1	30.9 ± 4.2	31.4 ± 4.2	0.000
Maternal schooling years	1775 (98.2)	15.3 ± 2.6	15.3 ± 2.5	15.3 ± 2.6	0.990
Maternal education	1787 (98.9)				
Diploma or under		528 (43.3)	255 (44.8)	783 (43.8)	0.889
Bachelor’s degree		504 (41.4)	234 (41.1)	738 (41.3)	
Master’s degree		168 (13.8)	73 (12.8)	241 (13.5)	
PhD/professional qualification		18 (1.5)	7 (1.2)	25 (1.4)	
Household income in CNY	1791 (99.1)				
< 20,000		431 (35.3)	215 (37.8)	646 (36.1)	0.031
< 30,000		264 (21.6)	148 (26)	412 (23)	
< 40,000		168 (13.7)	62 (10.9)	230 (12.8)	
< 50,000		67 (5.5)	17 (3)	84 (4.7)	
> 50,000		33 (2.7)	17 (3)	50 (2.8)	
Other		15 (1.2)	3 (0.5)	18 (1)	
Prefer not to say		244 (20)	107 (18.8)	351 (19.6)	
Maternal social class	1713 (94.8)				
IV & V		358 (30.7)	172 (31.5)	530 (30.9)	0.762
III		375 (32.1)	181 (33.2)	556 (32.5)	
I & II		434 (37.2)	193 (35.3)	627 (36.6)	
Infant gestation (weeks)	1783 (98.7)	38.8 ± 1.5	38.8 ± 1.4	38.8 ± 1.5	0.740
Infant gender	1803 (99.8)				
Male		645 (52.5)	279 (48.6)	924 (51.2)	0.125
Female		584 (47.5)	295 (51.4)	879 (48.8)	
Infant age (months)	1807 (100)	10.9 ± 3.1	6.6 ± 1.4	9.5 ± 3.3	0.000
Total No. of children in household	1794 (99.3)				
more than one child		454 (37.1)	211 (37)	665 (37.1)	0.945
only one child		769 (62.9)	360 (63)	1129 (62.9)	
Current accommodation	1631 (90.3)				
Bungalow		5 (0.5)	4 (0.8)	9 (0.6)	0.000
T-apartment without garden		112 (10.2)	124 (23.4)	236 (14.5)	
T-apartment with garden		259 (23.5)	100 (18.9)	359 (22)	
B-apartment without garden		162 (14.7)	66 (12.5)	228 (14)	
B-apartment with garden		538 (48.8)	231 (43.7)	769 (47.1)	
Villa		26 (2.4)	4 (0.8)	30 (1.8)	

*Notes*: * *P* < 0.05. SD = standard deviation

^a^ T-apartment is a type of apartment building with around ten residents living on each floor

^b^ B-apartment is a type of apartment with less than three residents living on each floor

Maternal age refers to the age at delivery

**Table 2:** Maternal birth experience and COVID impact during lockdown, according to whether they **delivered before or during the lockdown**

N(%)	***N*** (%)	Before	During	Total	***P*** value
		(***N*** = 1231)	(***N*** = 576)	(***N*** = 1807)	
Living status during the lockdown	1774 (98.2)				
With husband		409 (33.7)	221 (39.5)	630 (35.5)	0.168
With husband & parents		310 (25.5)	136 (24.3)	446 (25.1)	
With husband & parents-in-law		481 (39.6)	199 (35.5)	680 (38.3)	
Alone		9 (0.7)	2 (0.4)	11 (0.6)	
Other		5 (0.4)	2 (0.4)	7 (0.4)	
COVID impact on purchasing daily necessities	1792 (99.2)				
Great		106 (8.7)	57 (10)	163 (9.1)	0.014
Moderate		433 (35.5)	232 (40.6)	665 (37.1)	
Little		375 (30.7)	177 (30.9)	552 (30.8)	
No		306 (25.1)	106 (18.5)	412 (23)	
COVID impact on household income	1797 (99.4)				
Great		144 (11.8)	75 (13.1)	219 (12.2)	0.013
Moderate		466 (38.1)	246 (42.9)	712 (39.6)	
Little		297 (24.3)	143 (25)	440 (24.5)	
No		317 (25.9)	109 (19)	426 (23.7)	
COVID impact on birth location	1788 (98.9)				
Yes		58 (4.8)	61 (10.7)	119 (6.7)	0.000
No		1161 (95.2)	508 (89.3)	1669 (93.3)	
Birth location	1806 (99.9)				
National hospital		1177 (95.7)	552 (95.8)	1729 (95.7)	0.592
Private hospital		49 (4)	24 (4.2)	73 (4)	
Oversea hospital		3 (0.2)	0 (0)	3 (0.2)	
Home		1 (0.1)	0 (0)	1 (0.1)	
Mode of delivery	1802 (99.7)				
Vaginal		744 (60.6)	359 (62.4)	1103 (61.2)	0.656
Vaginal induced		38 (3.1)	22 (3.8)	60 (3.3)	
Planned caesarean		300 (24.4)	133 (23.1)	433 (24)	
Emergency caesarean		145 (11.8)	61 (10.6)	206 (11.4)	
Skin-to-skin contact after delivery	1804 (99.8)				
Yes		1184 (96.3)	555 (96.7)	1739 (96.4)	0.648
No		46 (3.7)	19 (3.3)	65 (3.6)	

*Note*: * P < 0.05

**Table 3:** Infant feeding practice, COVID impacts, and relevant feeding supports of subjects, according to whether they delivered before or during the lockdown

N (%)	***N*** (%)	Before	During	Total	***P*** value
		(***N*** = 1231)	(***N*** = 576)	(***N*** = 1807)	
Feeding intention before delivery	1800 (99.6)				
Exclusive breastfeeding		1014 (82.8)	485 (84.2)	1499 (83.3)	0.660
Formula feeding		31 (2.5)	13 (2.3)	44 (2.4)	
Mixed feeding		120 (9.8)	47 (8.2)	167 (9.3)	
No plans		59 (4.8)	31 (5.4)	90 (5)	
Infant feeding at 6-month	1800 (99.6)				
Mostly breastfeeding		746 (60.9)	392 (68.1)	1138 (63.2)	0.014
Formula feeding		79 (6.5)	32 (5.6)	111 (6.2)	
Mixed feeding		399 (32.6)	152 (26.4)	551 (30.6)	
Change in feeding intention and actual feeding for 6 month	1806 (99.9)				0.013
Yes, change		366 (29.8)	139 (24.1)	505 (28)	
No change		864 (70.2)	437 (75.9)	1301 (72)	
Current infant feeding	1806 (99.9)				
Mostly breastfeeding		88 (7.2)	210 (36.5)	298 (16.5)	0.000
Breastfeeding + solids		397 (32.3)	150 (26)	547 (30.3)	
Formula feeding		38 (3.1)	15 (2.6)	53 (2.9)	
Formula feeding plus solid		261 (21.2)	43 (7.5)	304 (16.8)	
Mixed milk feeding		44 (3.6)	42 (7.3)	86 (4.8)	
Mixed feeding plus solids		402 (32.7)	116 (20.1)	518 (28.7)	
COVID impact on original infant feeding	1796 (99.4)				
Great		66 (5.4)	30 (5.3)	96 (5.3)	0.036
Moderate		230 (18.8)	140 (24.5)	370 (20.6)	
Little		246 (20.1)	115 (20.1)	361 (20.1)	
No		683 (55.8)	286 (50.1)	969 (54)	
Received enough help with BF from professionals	1804 (99.8)				
Yes		1170 (95.3)	557 (96.7)	1727 (95.7)	0.163
No		58 (4.7)	19 (3.3)	77 (4.3)	
Received enough help with BF from family	1804 (99.8)				
Yes		1164 (94.8)	553 (96)	1717 (95.2)	0.260
No		64 (5.2)	23 (4)	87 (4.8)	
Enough support with own health	1693 (93.7)				
Yes		1048 (90.5)	488 (91.2)	1536 (90.7)	0.638
No		110 (9.5)	47 (8.8)	157 (9.3)	
Contact with a health professional (per week)	1783 (98.7)				
Never		756 (62.3)	347 (61)	1103 (61.9)	0.010
1–3 times		371 (30.6)	166 (29.2)	537 (30.1)	
4–5 times		29 (2.4)	31 (5.4)	60 (3.4)	
Daily or more		58 (4.8)	25 (4.4)	83 (4.7)	
Chat in a breastfeeding support group (per week)	1785 (98.8)				
Never		539 (44.4)	228 (40)	767 (43)	0.081
1–3 times		476 (39.2)	231 (40.5)	707 (39.6)	
4–5 times		93 (7.7)	41 (7.2)	134 (7.5)	
Daily or more		107 (8.8)	70 (12.3)	177 (9.9)	
Who supports infant feeding? (multiple choices allowed)					
Partner	1791 (99.1)	774 (63.5)	392 (68.5)	1166 (65.1)	0.037
Parent	1791 (99.1)	691 (56.7)	301 (52.6)	992 (55.4)	0.107
Parent In-laws	1791 (99.1)	557 (45.7)	260 (45.5)	817 (45.6)	0.925
Health professional	1791 (99.1)	551 (45.2)	207 (36.2)	758 (42.3)	0.000
Friend/relative	1791 (99.1)	356 (29.2)	125 (21.9)	481 (26.9)	0.001
Breastfeeding group	1791 (99.1)	386 (31.7)	164 (28.7)	550 (30.7)	0.200
Online support	1791 (99.1)	243 (19.9)	82 (14.3)	325 (18.1)	0.004
Other	1791 (99.1)	14 (1.1)	2 (0.3)	16 (0.9)	0.094
No support	1791 (99.1)	39 (3.2)	7 (1.2)	46 (2.6)	0.014

*Notes*: * P < 0.05. For multiple choices question, we compared each choice for DL & BL using the Chi-square. For single choice question we used the Chi-square and Mann-Whitney test for all categories

During the survey design, we calculated the expected sample size using the following function [17]:

$$n={\left[\frac{Z_{\alpha}\sqrt{\left({p}_1+{p}_2\right)\left(1-{p}_1+1-{p}_2\right)/2}+{z}_{\beta}\Big)\sqrt{p_1\left(1-{p}_1\right)+{p}_2\left(1-{p}_2\right)}}{p_1-{p}_2}\right]}^2$$

Based on previous literature, we estimated that the breast-feeding rate for first 6 months was 50% [18] and the estimated rate difference was 8% (p2 to be 58%), with statistical level α = 0.05 and power = 0.8 (1-β), two-sided test, the total sample size should be at least 1800.

Ethical approval was obtained from the Beijing Children’s Hospital Research Ethics committee (2020-Z-102).

### Content of the survey

The questionnaire was adapted from one intended for use in the UK, namely the COVID-19 New Mum Study [9, 19] with translation into Chinese. A repeated forward–backward translation procedure was adopted. The translated Chinese version was produced by a native Chinese speaker on the team and refined by a BCH paediatrician with clinical experience of infant feeding. Then the Chinese version was translated back into English and compared to the original English version. The survey comprises of four sections: 1) demographic characteristics of the participants; 2) maternal birth experiences and infant feeding; for the evaluation of infant feeding method, mothers were asked about the method of milk feeding used during the first 6 months (“what is the source of milk you used for feeding for the first six months after birth?”). The response options were exclusive breastfeeding, mixed feeding or formula-feeding. However, given that a strict definition of exclusive breastfeeding was not provided, and the question did not ask specifically about complementary foods, the exclusive breastfeeding category was renamed as ‘mostly breastfeeding’ (MBF) for the analyses, reflecting the majority source of the infant’s milk intake during this period.; 3) Maternal daily life and supports availables; and 4) the impact of COVID-19 on maternal mental health and life patterns. Detailed content is outlined in the appendix and can also be found in a previous publication using the same questionnaire [9, 20].

### Statistical analyses

For aim (1), we conducted descriptive analysis for mother’s background characteristics. For continuous variables (maternal age, maternal schooling year, infant gestational age and infant age), the mean (standard deviation, SD) was estimated for the eligible sample and for women who delivered before/during lockdown period, and independent t-test was used to compare the groups. For binary or categorical variables, frequency tables and cross tabs were applied to estimate the N (%) in each cell with chi-square test used to estimate the statistical difference.

For aim (2), to compare the distribution of mother’s birth experiences, infant feeding practices, COVID lockdown impact and support received among mothers delivered before/during lockdown, we also applied mainly cross tabs and chi-square to test the statistical difference.

Finally for aim (3), in order to investigate the determinants of maternal feeding practice for the first 6-months (breastfeeding or other feeding approaches) and to estimate whether lockdown had an impact on maternal feeding practice after controlling for other determinants, we first conducted univariate analysis (independent t-test or chi-square test) to explore the potential determinants (maternal SES backgrounds, received breastfeeding support, and delivery status) for maternal feeding practice. Then we applied logistic regression to estimate the relationship between maternal feeding practice during the first 6 months and delivered before/during the lockdown, with adjustment for covariates including maternal SES backgrounds and living conditions, delivery conditions, health and infant feeding support and COVID lockdown impact. Logistic regression was performed based on the Directed Acyclic Graph (DAG, eFigure 1). Adjusted odds ratio (OR) and 95% confidence intervals (CI) are presented, and *P* < 0.05 was considered as statistically significant. All statistical analyses were conducted using SPSS version 22.0 (IBM., Armonk, NY, USA).

## Results

### Demographic characteristics of the participants

From 1st August to 31st October 2020, 1807 mothers completed the questionnaire. All participants were married. At the time of survey completion, mean maternal age at delivery was 31.4 ± 4.2 years, and mean infant age 9.5 ± 3.3 months (range 1–18 months). The background characteristics are shown in Table 1. There were 576 (31.9%) and 1231 (68.1%) mothers who gave birth during the lockdown (DL) and before the lockdown (BL), respectively. Maternal education and infant gestation did not differ between the BL and DL groups. Infant age was higher as expected in BL (10.9 ± 3.1 months) than in DL (6.6 ± 1.4 months) (*p* < 0.001) due to the experimental design of the study. Mothers in the BL group were also older at delivery than those in the DL group (*p* < 0.001) and more were in the lowest category for education and household income compared to the DL group (*p* < 0.05).

### Birth experiences and daily life

For DL and BL mothers, 62.4 and 60.6% were vaginally delivered and the post-birth skin-to-skin contact was 96.7 and 96.3% respectively; with no significant differences between groups. Over 95% of the women delivered at a national hospital. Notably, significantly more mothers in DL groups reported that the COVID-19 had impact on their birth location, compare to BL mothers (10.7% vs. 4.8%, *p* < 0.001). Moreover, compared to BL mothers, significantly more women in the DL group considered COVID-19 to have had a moderate-to-high impact on their household income (49.9% vs. 56%, *p* = 0.013) and on the convenience of purchasing daily necessities (44.2% vs. 50.6%, *p* = 0.014) (Table 2).

### Infant feeding during the lockdown

Infant feeding intentions did not differ between DL and BL groups (Table 3). Among DL women, 29.8% reported a moderate-to-high impact of COVID-19 on their infant feeding practice, compared to 24.2% in the BL group (*p* = 0.036). In the DL group, significantly more mothers reported 6-month MBF compared to the BL mothers (68.1% vs. 60.9%, *p* = 0.014). As expected, given the younger age of the infants of DL mothers, a significantly higher proportion of mothers were MBF (36.5%) compared to BL mothers (7.2%) at the time of completing the questionnaire, while a greater proportion of older infants born BL also consumed solid foods (Table 3).

### Infant feeding support during the lockdown

Overall, more than 90% of mothers in both BL and DL groups reported they had received enough supports during the lockdown, by answering “yes” to the question “Did you received enough help with breastfeeding from professionals/family?” and “Did you received enough support with your own health?” (Table 3), suggesting that mothers who had given birth during the pandemic may have received same overall support than those who had given birth before. The main reported sources of infant feeding support (‘Where do you get support with infant feeding?’) were the partner (65.1%), followed by parents (55.4%), and parents-in-law (45.6%), health professionals (42.3%), breastfeeding support groups (30.7%), friends and family (26.9%), and online support (18.1%). A significantly higher proportion of DL mothers reported having received support from their partner compared to the BL group, whilst a larger proportion of BL mothers received support from health professional, friends and relatives, online support sources or reported no support during the pandemic (Table 3).

No difference was found between BL and DL mothers regarding the frequency of participating in a breastfeeding support group. Significantly more DL women reported having contacted health professionals more than four times a week (19.5%), compared to those from the BL group (16.5%).

### Predictors of infant feeding during the first 6-months

Results of the logistic regression show that MBF during the first 6 months was positively predicted by delivery DL versus BL (OR1.43, 95%CI 1.08, 1.90). Positive predictors of MBF (Table 4) also included younger maternal age, infant feeding discussion in an online support group more than four times a week, and receiving support from friends or relatives versus no support.

**Table 4:** Univariate and multivariable logistic regression for determinants of whether or not MBF for the first 6 months

6-month mostly breastfeeding rate	N (%)	Univariate analysis			Multivariable Logistic Regression			
		Non-MBF	MBF	***P*** value	95% CI for OR			
		(***N*** = 662)	(***N*** = 1138)	(T = 1800)	OR	Lower	Upper	***P*** value
Infant born before/during lockdown?	1800 (100)			0.003				
born before lockdown		479 (39.1)	746 (60.9)		ref			
born during lockdown		184 (31.9)	392 (68.1)		1.43	1.08	1.90	0.012
Maternal age (years)^a^	1764 (98)	31.8 ± 4.3	31.2 ± 4.1	0.000	0.96	0.93	0.99	0.011
Total years of maternal full-time education	1769 (98.3)	15.4 ± 2.5	15.3 ± 2.6	0.620	1.02	0.96	1.07	0.618
Household annual income in CNY	1417 (78.7)			0.781				
< 200,000		239 (37.2)	404 (62.8)		ref			
200,000-300,000		161 (39.2)	250 (60.8)		0.94	0.69	1.29	0.700
> 300,000		135 (37.2)	228 (62.8)		1.36	0.96	1.93	0.089
Maternal social class ^b^	1706 (94.8)			0.233				
IV & V		185 (35.1)	342 (64.9)		ref			
III		197 (35.6)	357 (64.4)		1.08	0.76	1.53	0.666
I & II		247 (39.5)	379 (60.5)		0.77	0.55	1.07	0.113
Building living ^c^	1627 (90.4)			0.204				
Bungalow		5 (55.6)	4 (44.4)		ref			
T apartment without garden		79 (33.5)	157 (66.5)		3.82	0.66	22.13	0.134
T apartment with garden		119 (33.2)	239 (66.8)		4.75	0.83	27.16	0.080
B apartment no garden		83 (36.4)	145 (63.6)		3.67	0.64	21.21	0.146
B apartment with garden		302 (39.4)	465 (60.6)		2.82	0.50	15.84	0.240
Villa with garden		9 (30)	21 (70)		3.53	0.50	25.18	0.208
whether had support from families/friends after birth	1797 (99.8)			0.360				
No		36 (41.4)	51 (58.6)		ref			
Yes		625 (36.5)	1086 (63.5)		1.95	1.06	3.59	0.032
Enough infant feeding support?	1784 (99.1)			0.053				
not enough		23 (50)	23 (50)		ref			
enough		628 (36.1)	1111 (63.9)		1.44	0.69	3.03	0.334
Enough support with own health?	1686 (93.7)			0.060				
not enough		68 (43.3)	89 (56.7)		ref			
enough		548 (35.8)	982 (64.2)		1.42	0.90	2.24	0.135
Contact with a health professional (/week)	1776 (98.7)			0.050				
Never		428 (38.9)	673 (61.1)		ref			
1–3 times/week		183 (34.3)	351 (65.7)		1.05	0.77	1.43	0.759
4+ times/week		43 (30.3)	99 (69.7)		1.12	0.65	1.94	0.684
Discuss health issues in mothers’ group online (/week)	1778 (98.8)			0.040				
Never		305 (39.9)	459 (60.1)		ref			
1–3 times/week		251 (35.6)	455 (64.4)		1.23	0.91	1.68	0.177
4+ times/week		99 (32)	210 (68)		1.66	1.09	2.53	0.019
Mode of delivery	1796 (99.8)			0.000				
Vaginal		387 (33.4)	772 (66.6)		ref			
Caesarean		275 (43.1)	363 (56.9)		0.77	0.59	1.01	0.059
whether COVID impacted birth location	1782 (99)			0.584				
Yes		41 (34.5)	78 (65.5)		ref			
No		615 (37)	1049 (63)		1.67	0.96	2.90	0.067
COVID impact on purchasing daily necessities	1785 (99.2)			0.355				
Moderate-great impact		294 (35.6)	532 (64.4)		ref			
Little or no impact		362 (37.7)	598 (62.3)		0.80	0.61	1.04	0.096

*Notes*: *MBF* Mostly breastfeeding, *CI* confidence interval, *DL* mothers who delivered during the lockdown, *BL* mothers who delivered before the lockdown. Factors controlled for included maternal socio-economic status (education, income, social class), living conditions, cohabitation with family members (“Who did you live with during the lockdown?”), gestational age, health advice-seeking behaviour (“Frequency of discussing with health professionals/chatting in breastfeeding support group”), support (“Did you receive enough support from health professionals/family members?”), and the impact of the COVID-19 lockdown on maternal daily life

^a^ Younger maternal age as reference

^b^ Class IV&V mainly refer to manual labor jobs which don’t require high education experience; Jobs in class III & II require certain training, certifications, licenses and degree to qualify; Jobs in class I involve professional careers which require advanced degree, high-end skills or expertise

^c^ T-apartment is a type of apartment building with around ten residents live in each floor; and B-apartment is a type of apartment with less than three residents live in each floor

### Comments

#### Summary of the results

Our findings show that, despite the difficulties imposed by the pandemic, hospital facilities in Beijing continued to implement measures based on WHO guidelines [8, 21, 22]. No significant differences were found in maternal delivery method, postnatal skin-to-skin contact, or breastfeeding supports between BL and DL mothers. However, significant differences were found in perceived lockdown impact on household income, convenience of buying daily necessities, changes of birth location, feeding plan, and current feeding methods between BL and DL mothers. The MBF at 6-months was predicted by delivery during the lockdown period, younger maternal age, getting support from friends or relatives, and discussing health issues in online groups more than four times a week.

#### Maternal birth experience, infant feeding practice and support

A significantly higher number of mothers in DL group reported their intended birth location had been affected by the pandemic, reflecting mothers who originally planned to give birth at a private hospital but were changed to a public hospital or vice versa. Some may have chosen a private hospital in order to reduce contact with others, whereas some mothers may have believed that a public hospital could provide better treatment than a private maternity hospital should they become infected; however, we did not record this information as part of the survey.

At 6 months, significantly more DL mothers were mostly breastfeeding compared to BL mothers,

suggesting that the lockdown measures may have indirectly promoted breastfeeding, perhaps by allowing mothers to spend more time with their babies, or feeling less pressure due to fewer visitors during the lockdown [16]. A similar finding was reported in the COVID-19 New Mum Study in the UK [9], where 13% of women reported changes to feeding method due to the lockdown; a higher frequency and longer duration of breastfeeding was observed in 30 and 17% women who were breastfeeding during the pandemic, reflecting more time spent at home as well as a greater contribution to childcare from themselves and partners during the lockdown period. However, contrasting findings were observed in another cross-sectional study, which reported a slight decrease in breastfeeding in 903 mothers with infants ages 0–12 months during the COVID-19 lockdown in Thailand [23]. Thus, the impact of lockdown measures on infant feeding might differ depending on access to support and the particular circumstances experienced by individual mothers. Indeed, our results suggest that although DL and BL mothers reported to have received sufficient support in general, significant differences were observed between groups regarding the exact person who provided the main supports. Nevertheless, both peer and professional support would be important for the success of breastfeeding [24].

Research on breastfeeding support has consistently identified the husband or partner as an important source of support for women, with influence on four aspects in particular: breastfeeding decisions, assistance at first feeding, assistance during breastfeeding, and risk factors for bottle feeding [13]. Our study found that women from both groups considered their partner as the greatest source of support for infant feeding, consistent with the UK New Mum study [9] . Compared to BL mothers, DL mothers received even more support from their partners, possibly reflecting the “work from home” measures in place during the lockdown, which increased the time husbands could spend with their wives. As suggested by Vazquez et al. [9], husbands who contribute more to childcare during this exceptional period may represent a valuable source of support for women, especially for those with limited access to friends or relatives who would provide this support under normal circumstances. Hence. Partner’s supports should be emphasized in breastfeeding education activities.

According to Chinese tradition, mothers normally adhere to a month long “confinement period” after birth. Although an increasing number of mothers use a confinement centre where they can receive care and professional support, most centres were closed during the lockdown period; this may have led to differences in the confinement experience for mothers in the DL and BL groups. However, a recent study found that the 1- and 6-month EBF rate did not differ between mothers who chose a confinement centre and mothers who stayed at home (37% vs. 42%, *p* = 0.5) [25]. Whilst more studies are needed to confirm this finding, we suggest that mothers in the DL group may have received more support from their partner, so the total support received by the DL and BL mothers might have been the same.

#### Predictors of infant feeding during the lockdown

Mothers who delivered during the lockdown were more likely to mostly breastfeed their infant during the first 6 months. This may reflect the increased time available for both mothers and their partners to spend with the infant and the breastfeeding support provided during the lockdown. Higher rates of MBF were also observed among mothers who delivered at a younger age. This result is consistent with a previous study in Zhejiang, China, which indicated that EBF was positively related to younger maternal age [11]. However, this contrasts with studies in many high-income countries which indicated that older maternal age was associated with a higher rate of breastfeeding initiation and the duration of EBF [14, 26, 27]. This may reflect social and cultural factors, and emphasizes the importance of considering these factors when developing health policy to promote breastfeeding. In recent years, EBF has become a “new fashion” in several modern cities in China, including Beijing, Shanghai, and some large cities in Zhejiang province, possibly reflecting the increasing number of Chinese female celebrities sharing their EBF experiences and healthy mother-infant relationships through vlogs or posts. Young women from these cities are keen to follow this new fashion and are very proud if they successfully EBF to 6 months. Such kind of “celebrity effects” can be suggested in future breastfeeding promotion campaigns. Additionally, our results suggest that discussing infant feeding in a feeding support group more than four times a week, getting support from friends or relatives compared to no support positively predicted MBF in the first 6-months, reflecting the vital role of infant feeding support in promoting breastfeeding for mothers.

It worth to notice that for the feeding method at 6-months, we re-coded the original answer “EBF” to “MBF” for the question “what is the source of milk you used for feeding for the first six months after birth?”, considering we did not provide a specific definition of EBF and, whilst nurses were available to help mothers if they had any queries, some mothers may not have asked for clarification. Moreover, the question in the survey referred to milk feeding but did not specifically ask about the use of complementary foods before 6 months. However, the 6 months MBF rates in the survey population still appear relatively high compared to previous studies in China (60.6 and 71.6% mothers in the BL and DL group) [28]. This might reflect the inclusion criteria for the study which specified that participants must have breastfed their infant during the lockdown. Besides, rates of EBF vary across provinces and cities in China. According to the data published in 2013, the EBF rate for 14,539 children from 30 provinces in China was 20.8% [29], whilst a large cohort study conducted in Zhejiang province involving 42,550 children reported a 3-to 5 month EBF rate of 51.3% for girls and 46.8% for boys [28]. However, in that cohort, EBF ≥ 6 months was defined as self-reported EBF at each of the 3 clinical visits (1, 3, and 6 months), thus the EBF rate may have been over-estimated.

#### Strengths and limitations

To our knowledge, this is the first cross-sectional study investigating infant feeding practices and potential predictors of infant feeding during the COVID-19 pandemic in China. Using an adapted version of questionnaire that has been used in the COVID-19 New Mum Study [9, 19] in the UK makes the results comparable between countries. However, the study has some limitations. Firstly, although Beijing is a metropolitan city with 20 million residents from different regions of China [30], study participants are not representative of all new mothers in China; the significantly higher gross domestic product per capita in Beijing results in a higher level of family income and education of the population [31], which may partly explain the higher MBF rate in this study [32]. However, considering that the duration of lockdown and the type of lockdown measures varied between provinces and cities in China, it would have been difficult to compare the infant feeding outcomes between cities even in a national survey. Secondly, there may be recall bias since at the time of survey completion lockdown measures had just ended in Beijing. Moreover, the questionnaire used in the present study was originally designed by our research team at the very early stage of the COVID-19 pandemic, hence, there could be potential confounding factors that did not been identified and included in the questionnaire beforehand. Last but not least, we did not clearly define EBF in our questionnaire so; consequently, we relabeled this variable as ‘mostly breastfeeding (MBF)’.

## Conclusions

Our findings show that the pandemic and lockdown measures in Beijing affected mothers’ planned birth location and breastfeeding intention. However, despite difficulties imposed by the pandemic, feeding support was generally well preserved for mothers who delivered a baby during the lockdown, reflected in high rates of mostly breastfeeding in the first 6 months. This highlights the importance of breastfeeding support both from peers and from professionals during public health emergencies. More importantly, our results suggest that by allowing parents to spend more time to contribute to childcare during the lockdown period, the breastfeeding may be promoted. This may be important not only during public health emergencies but also under normal circumstances.

## Supplementary Information

**Additional file 1: eFigure 1**. Directed acyclic graph of the factors related to infant feeding at first 6 months of birth.

**Additional file 2.** .

## Data Availability

The datasets used and/or analysed during the current study are available from the corresponding author on reasonable request.

## Abbreviations

COVID-19: coronavirus disease 2019
SARS-CoV-2: severe acute respiratory syndrome coronavirus 2
DL: during the lockdown
BL: before the lockdown
MBF: mostly breastfeeding
EBF: exclusive breastfeeding
DAG: directed acyclic graphs
SD: standard deviation
OR: odds ratio
CI: confidence intervals
