# Supplementary figures and images for: The impact of the Covid-19 pandemic on maternal delivery experiences and breastfeeding practices in China: data from a cross-sectional study
Source: BMC Pediatr. 2022 Feb 24;22:104. doi: 10.1186/s12887-022-03155-y (PMC8866109; doi:10.1186/s12887-022-03155-y)

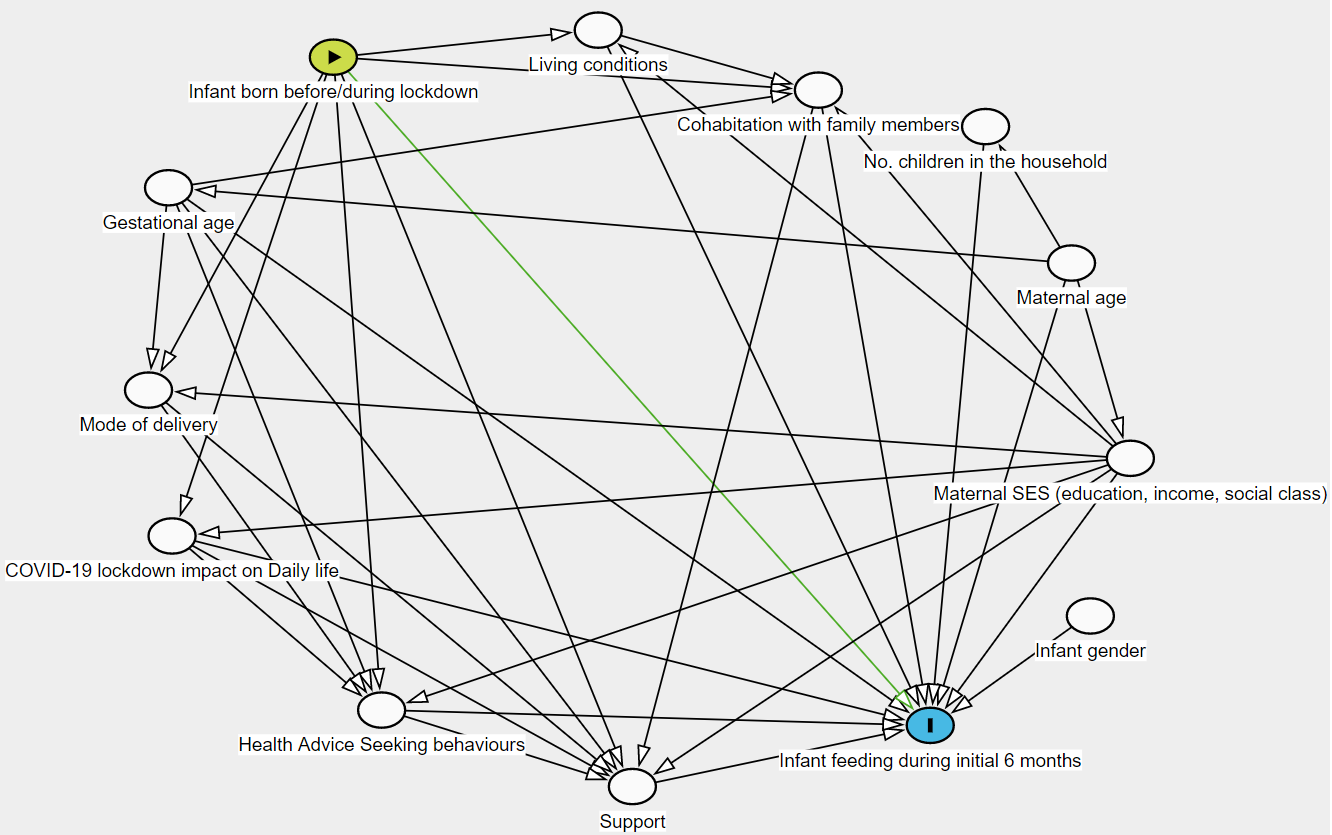

Supplement: Supplementary file 1 — Additional file 1: eFigure 1. Directed acyclic graph of the factors related to infant feeding at first 6 months of birth. [file 12887_2022_3155_MOESM1_ESM.tif]
